# Supplementary material for: Research Methods for the Analysis of Visual Emotion Cues in Animals: A Workshop Report
Source: Animals (Basel). 2025 Oct 29;15(21):3142. doi: 10.3390/ani15213142 (PMC12609227; doi:10.3390/ani15213142)
Supplement: Supplementary file 1 [file animals-15-03142-s001.zip › animals-3932666-supplementary.pdf]

Supplementary Information for:

Conference Report

Research Methods for the Analysis of Visual Emotion Cues in Animals: A Workshop Report

Catia Correia-Caeiro[1,2,3,*], Anna Zamansky[4], Sabrina Karl[5], Annika Bremhorst[4,6,7,*]

1 Human Biology & Primate Cognition, Institute of Biology, Leipzig University, 04103 Leipzig, Germany
2 Comparative Cultural Psychology, Max Planck Institute for Evolutionary Anthropology, 04103 Leipzig, Germany
3 Center for the Evolutionary Origins of Human Behavior, Kyoto University, Inuyama, 484-8506, Japan
4 Tech4Animals Lab, Department of Information Systems, University of Haifa, Haifa 31905, Israel; annazam@gmail.com (A.Z.); annikahubercdl@gmail.com (A.B.)
5 Independent Researcher, Vienna, Austria; sabrina.karl82@googlemail.com
6 Dogs and Science, 8142 Uitikon-Waldegg, Switzerland
7 Department of Clinical Veterinary Science, Clinical Anesthesiology, Vetsuisse Faculty, University of Bern, 3012 Bern, Switzerland
* Correspondence: catia_caeiro@hotmail.com, annikahubercdl@gmail.com

Table S1: Speakers, talks, and key points from the First International Workshop on Research Methods in Animal Emotion Analysis (RM4AEA).

Speaker and affiliation	Talk title	Key points speakers' talks
Bridget Waller Nottingham Trent University	Introduction to Facial Behaviour and how to measure it using FACS	● Assumed one to one relationship between internal state (feeling/emotion) and behaviours (expressions).<br>● Issues: attribute emotions incorrectly, particularly in non-verbal individuals, we are isolating the visual cues from the social interactions when looking only at the sender.<br>● The same facial expression appearance in different species is used in different contexts (different meanings, e.g., happy human smile vs fearful bared teeth display in macaques).<br>● Facial Action Coding System (FACS) applications: identify homologues between species, identify subtle variations in the same facial expression, how domestication impacted the facial expressions, how complex are communication systems.<br>● Facial expressions aid others in predicting the sender's behaviour and have a role in social bonding: the more complex the facial communication is, the more it contributes to social bonding, and the more complex a society is.<br>● Humans have individual differences in how they use facial expressions and these are stable throughout life.
Catia Correia Caeiro, Leipzig University / MPI-EVA / Kyoto University	How to use FACS to understand animals, from dogs to primates?	● Faces are always processed in our brains in terms of emotion and in a global way, which means we cannot see the actual behaviour of the face and we miss subtle details.<br>● FACS measures what is happening on the face.<br>● FACS is independent of emotion, meaning, and context.<br>● All AnimalFACS development follow the same 3-step methodology: definition of the facial muscle plan, analysis of spontaneous facial behaviour, and classification of homologous movements into Action Units (AUs).<br>● All AnimalFACS are free and available at AnimalFACS.com.
Jumpei Matsumoto	MacaquePose: a novel data set for deep learning	● In order to study neuropsychiatric diseases in non-human primate models, with non-verbal individuals, we need quantitative and objective behavioural analysis.<br>● Objective measurements will also improve reproducibility of results.

University of Toyama	based for marker-less motion capture of macaque monkeys	● Motion capture of body movements provides information not only on normal healthy motor function, but also social and emotion information.<br>● MacaquePose is a markerless method developed based on DeepLabCut (DLC) that does not impact the individuals' behaviour.<br>● The performance of the network was close to human proficiency.<br>● The data set also works well with multi-animal pose estimation and other algorithms.
Pia Haubro Andersen Swedish University of Agricultural Sciences	The bumpy road towards automated recognition of facial expressions of horse pain	● Access to data sets on animal pain will have an enormous impact on animal welfare.<br>● 10 seconds a clinician looks at an animal is not enough to assess pain, we need automated ways of doing this.<br>● In horses, pain and stress facial expressions seem to overlap.<br>● It's difficult to publish work that says horses are in pain.<br>● Behavioural indicators are the most precise to assess pain.<br>● Stress and fear can increase or decrease the experience of pain.<br>● Behavioural assessment of pain is better than pain assessment - clinicians don't agree on pain assessment, even in situations that must cause a lot of pain because nerves are cut (e.g., castration).<br>● Grimace scales are hot topic for pain assessment, but none of them works.<br>● EquiFACS works better; AUs need to be selected, either on an arbitrary limit (5% more than normal or temporal co-occurrence).<br>● We found a prototypical pain and stress face in the horse with EquiFACS comprised by a cluster of AUs (not by single AUs).
Anjuli Barber, Dog Emotion Lab	At the end of their rope: Understanding dog emotions!	● We know a lot on how dogs discriminate and recognise human emotions, but we know a lot less about how dogs display emotion.<br>● Lateralisation of behaviours is becoming one of the most important approaches to the study of emotion in animals.<br>● We need a multi-modal approach to better assess emotion.<br>● The EMOMETER, an integrated toolbox to measure emotion functioning in dogs, includes behaviour (body/face expressions), physiology (cardiac and endocrine responses), lateralisation, body surface temperature, visuo-cognitive processes (e.g., perceptual processes), and skin conductance responses.

		● No links were found between paw preference and emotion in dogs, suggesting instead that paw preference is task-specific [96].<br>● Head tilts seem to be linked to type of auditory stimuli, but not breed appearance nor sociability, and does not seem to be linked to emotion either.<br>● Submissive grinning always left lateralised and seems to be linked to negative arousal in dogs.<br>● Heart rate and skin conductance correlate in arousing contexts.
Suresh Neethirajan, Wageningen University	Artificial Intelligence in Investigation of Farm Animals' Affective States	● There is no consensus on the definition of emotion and some authors don't even consider emotions of feelings, but for animal welfare we need that.<br>● We need to assess the fluctuating emotions and feelings of animals throughout time, which automated systems can help us do.<br>● Using video of free moving farm animals, we were able to assess emotion, but also intensity based on facial movements, by applying a number of different algorithms.<br>● We still need a lot of validation, and we need to use more multimodal approaches.<br>● Because of the lack of consensus, we need to pool together with physiological measurements.<br>● Pigs display more facial movements than cows, whilst for cows superficial temperature seems more relevant.
Annika Bremhorst, University of Bern/ University of Lincoln	Investigating facial expressions and their accuracy as potential emotion indicators in dogs	● In positive anticipation, dogs showed consistently one ear movement (EAD102 - Ears Adductor included in the DogFACS), similarly to a previous study.<br>● In frustration, several facial movements from DogFACS were also identified, namely Ears Flattener, Blink, Nose Lick, Lips Part, and Jaw Drop, similar to a previous study.<br>● In addition, four new movements were observed in the frustration condition: Ears downwards, Tongue Show, Lip Corner Puller, and Upper Lip Raiser.<br>● However, Upper Lip Raiser varied with the reward used, and hence it cannot be considered specific of the emotion.
Anna Zamansky, University of Haifa and	Investigating facial expressions in dogs:	● What can we put inside the classifier "black box" to discriminate between positive anticipation and frustration in dogs? A few approaches: FACS coding, other features, or do not use any input and let the machine discover what's different.<br>● Our results show it is very hard to automate the detection of AUs.

Annika Bremhorst, University of Bern/ University of Lincoln	opportunities for AI	● Maybe the machine does not need DogFACS, but it is useful to make the results explainable.
Sofia Broomé, KTH Royal Institute of Technology	Video-based recognition of different pain types in horses using deep learning	● We were the first team to use video sequences with a deep learning approach (i.e., the machine is learning spatiotemporal patterns).<br>● The system outperforms veterinarian experts.<br>● The AI models learned from pixels (no intermediate input).<br>● Humans are particularly bad at recognising non-pain, which machines do well.<br>● Domain transfer is possible between different types of pain in the horse.<br>● AI found features of pain not identified by humans.
Marie-Claire Pagano, Cameron Smith, Yu Guan and Lucy Asher, Newcastle University	Automated labelling of facial features in dogs using DeepLabCut	● Using DLC is user friendly and does not require large amounts of data, hundreds of labelled images is enough, as it uses transfer learning.<br>● The model was less accurate to label new dogs (test phase), due to video quality variation.<br>● The model will be made available after improvement.<br>● This is an easy to use and implement method to track dogs' facial movements.

Table S2: Discussion panel with the speakers (**Table S1**), questions, and key points from the First International Workshop on Research Methods in Animal Emotion Analysis (RM4AEA).

**Questions from the organisers**	**Panellist**	**Key points from panellists' answers**
1. What are the main challenges in measuring emotions?<br>2.How are animals different from humans in this aspect?<br>3.How do we know whether the behaviour is linked to emotion? (as in, not all behaviours are an emotion cue).	Suresh Neethirajan, Wageningen University	● Animals are different from humans, as humans can fake emotions, animals cannot.<br>● To know that a behaviour is linked to emotion we can use correlates between chemical responses and behavioural responses.
	Pia Haubro Andersen, Swedish University of Agricultural Sciences	● Problem with pain: no gold standards to assess it.<br>● What is a happy horse? - horse ethologists answer that is a calm content horse that enjoys being a horse.<br>● We need footage on neutral emotion to compare everything else to. In humans, there is a range of emotions that can be classified as calm/content.<br>● If I have a gold standard for emotion, can you have a gold standard for non-emotion?<br>● I would like to find some positive emotion cues in the horses, like it was found in dogs, but I don't seem to find any.<br>● It is important that we do not medicate horses that do not need pain medication, but are instead stressed and need environment changes, for example.
	Annika Bremhorst, University of Bern / University of Lincoln	● We talk about a baseline instead of a neutral, because we consider it to be low arousal and slightly positive valence (hence, not no emotion).
4. How critical are biases introduced by researchers when interpreting animal behaviour (e.g., anthropocentrism,	Bridget Waller, Nottingham Trent University	● How do we know that species react in the same way to different contexts, are we looking at species differences or temperament differences?<br>● The worry with AI is if we are imposing what we think are positively and negatively valenced situations, but how do we really know that? Is it easy to do that? Probably not, because it is difficult to do that, at least not in an anthropomorphic or anthropocentric way. That's why FACS is such a good tool,

anthropomorphism, cognitive biases) and in the data sets from which AI learns, and how can they be overcome?		because it's a bottom-up approach without assumptions. But we don't really know and it is a difficult question.<br>● Unsupervised approaches may introduce more biases than using DogFACS, as much as possible remove the judgements we have to make to identify the movement.<br>● But there may be other factors, such as body or context, or receiver responses.
	Catia Correia Caeiro, Leipzig University / MPI-EVA / Kyoto University	● One of the biggest concerns in this area is that we just assume a happy dog looks in a certain way, and then that's the stimulus we use for everything. But we don't really know how a happy dog looks like, we just assume it. We only know how a happy dog looks like if we add the context, we give food to the dog, we play with the dog.<br>● We can look into a dog's eyes and easily presume we know how they feel, but this is not scientific. That's why FACS helps skipping these biases.<br>● Humans have many biases even if we are careful, and with the AI we have the same issue, because the human is programming the AI, so biases will seep into it.
	Annika Bremhorst, University of Bern / University of Lincoln	● With FACS we have an opportunity to overcome the biases.<br>● We try to operationalise the emotions that we study, but still there can be some kind of biases we expect the animal to have.<br>● It is difficult to distinguish positive anticipation and frustration because they are related.<br>● We need to consider individual differences.<br>● Humans are creating and defining the situations where we collect the emotion cues.
	Suresh Neethirajan, Wageningen University	● For example, humans showing teeth may be positive, but in animals this often varies, may be pain, aggression, etc.<br>● Pain is subjective, depends on many factors, individual animals have different tolerance to pain, and different pain triggers are different.<br>● What we need is a benchmark, and once this is implemented we can develop the tool. We need multimodality for creating a much more improved version of AI detection.

	Pia Haubro Andersen, Swedish University of Agricultural Sciences	● There is an expectation bias with FACS: if a coder sees one AU related to pain, they start expecting and overcoding other AUs of pain - we yet have not this empirical evidence, but what Sofia showed us with the orthopaedic pain study is that it is so easy to see that the head movements are pain indicators, so better blind processes are needed.
5. How can we push the field of animal emotion analysis forward, provided the great interest this workshop has generated? 6. How can we further advance/support interdisciplinary exchange between animal/human behaviour scientists and computer scientists?<br>7. What are the challenges in such an exchange?	Bridget Waller, Nottingham Trent University	● What makes this field so exciting is its interdisciplinarity: not only areas of research, but also a really interesting combination of applied and fundamental sciences.<br>● When we first created the systems, the purpose was more theoretical questions, but it is great to see it in so many applied questions.<br>● There is great potential for funding because of this multidisciplinarity nature.
	Pia Haubro Andersen, Swedish University of Agricultural Sciences	● Some ethical challenges in sharing data, not only data privacy, but regarding what is on the films, and permissions granted from owners.<br>● If the pain is very mild, the machine cannot use it, which is controversial. Many journals want to have the data set published. It's hard to have sensitive images, people do not like to have these, although horses are dying with a lot of suffering, and this is not the same as putting a blood pressure cuff.<br>● If we could scrutinise a standard contract as how we handle the ethics, so people realise these data may not be shared with anyone. It is very dangerous to let this data freely available.
	Suresh Neethirajan, Wageningen University	● Producing a white paper, creating a consortium, setting up a platform, to look at standardising the benchmarks that are not yet available.
	Anna Zamansky, University of Haifa	● Using the power of multidisciplinarity, we can acquire more funding.<br>● We want to use this workshop and what we are creating here to advance the field.<br>● To automate FACS, we need a lot of AUs examples.

	Catia Correia Caeiro, Leipzig University / MPI-EVA / Kyoto University	● Automate FACS is essential, because the funding for projects mostly goes into the coding. So, we need computer scientists to help us doing this.

The emotion debate: Key concepts, considerations and recommendations in relation to animal emotion analysis

There is currently a heated debate on fundamental issues in the field of emotion research, including definitions and approaches to investigating this subject [97-99–4]. This debate becomes even more intense in non-human animals (henceforth animals) who cannot communicate verbally. At one end of this debate, it is argued that animals do not experience emotions like humans do due to lack of awareness, and because emotions are created through cultural and contextual factors. Conversely, at the opposite end, considering the vast neurobiological, physiological, and behavioural evidence, the parsimonious explanation is that it is highly likely that animals experience (at least some) emotions [97,100]. Other researchers acknowledge that animals likely experience emotions, albeit differently from humans, but that studying these emotions is impractical, suggesting instead that research should concentrate on observable communication cues, like facial expressions [101]. While we recognise the challenging nature of this research field and its ongoing conceptual and theoretical debate, we believe that developing and employing scientific and objective tools to measure correlates of emotion in animals is a worthwhile scientific endeavour.

Our operational definition of emotion

Emotions are short-lived internal states occurring in response to emotionally-competent stimuli (i.e., external/environmental cues with a certain configuration, context, and intensity, which trigger a multicomponent emotion response, [1,102]) that are detected (consciously or unconsciously) by an individual. Upon detection of these stimuli a neuro-hormonal response may be triggered (e.g., amygdala activation, release of Corticotropin-Releasing Factor (CRF, a neurotransmitter released during aversive events) [103,104]), which in turn produce (or are accompanied by) a range of internal and external changes in the individual, including cognitive (e.g., appraisal or biases), physiological (e.g., hormonal, cardiovascular), motor (e.g., muscular tension, flight locomotion), and behavioural (e.g., facial expressions, body postures, vocalisations) [1,105].

One of the advantages of this reinforcement-based definition is in their behavioural operationalisation using the concepts of “reward” and “punishment” as something which animals which will work to, respectively, access or avoid. This allows emotions to be defined in a way that avoids circular argument and helps with establishing ground truth. For the purpose of this work, we view “rewards” and “punishments” more generally as external/environmental emotion-competent stimuli that are perceived by the individual as positive and negative, respectively.

Examples of multi-components of emotions

Functional magnetic resonance imaging (fMRI) studies can directly measure neural activation in response to emotionally-competent stimuli, (e.g., [106-109]), and cognitive bias experiments can measure changes in the perception of a manipulated stimulus, indirectly measuring the emotion of the individuals (for a review, see [110]). Moreover, physiological measurements of heart rate variability [111,112] and salivary hormonal samples [113,114] can be collected when individuals are presented with a stimulus, indicating how they respond internally to that stimulus. Finally, motor and behavioural changes, such as facial [8,16,17] or bodily expressions [25,115] can be quantified by using diverse methods and devices, ranging from external activity monitors [116], motion sensors [117], various observational techniques [118], and more recently, even automated computer systems [119].

Example of a diagnostic accuracy assessment to evaluate an emotion indicator potential

Even when behaviours accompany a specific emotion consistently across different situations, their reliability as potential indicators still needs to be evaluated, with additional measures, such as the ones provided by diagnostic accuracy assessments. Originally used in medical research to evaluate test performance in identifying the presence or absence of a disease, diagnostic accuracy assessment can also be applied to the study of emotion indicators [17].

For instance, dog facial behaviours that accompany positive anticipation and frustration exhibited low accuracy as single indicators of emotions, even though these were behaviours that were observed in different circumstances in which dogs were assumed to experience positive anticipation and frustration [17]. This also highlights the need to explore the multi-modality of emotion behaviour correlates, where the combination of various facial and/or bodily behaviours may enhance accuracy [81]. However, there is currently a lack of systematic and comprehensive research in this area, and further investigation is needed to improve our understanding of diagnostic accuracy in identifying reliable emotion indicators.

Recommendations and remarks for the interpretation of emotion correlates

The following three points serve as guidance for researchers and practitioners regarding emotion correlates in animals.

1) Empirical basis: Assumptions about (interpreting) animal emotions based solely on behaviour without empirical evidence, which is currently limited, should be avoided. Hence, we recommend to:

- Conduct more systematic studies to investigate behavioural correlates in specific emotion contexts.
- Avoid prematurely labelling a behaviour as an emotion indicator solely based on its occurrence in a particular emotion situation (it may be rather related to contextual factors, including motivations, than the underlying emotion itself).
- Perform rigorous analyses, such as diagnostic accuracy assessment, to evaluate the potential of behavioural correlates as reliable emotion indicators.

2) Triangulation of information: To enhance the accuracy of emotion assessments, we recommend to triangulate behavioural expressions with complementary sources of information to establish a more robust foundation for interpreting animal emotions [120]. In addition, we also recommend to:

- Consider multiple modalities, such as vocalisations, body posture, facial movements, and context cues, in order to develop a comprehensive understanding.
- Evaluate behavioural expressions within the situational context, encompassing environmental conditions, social interactions, and specific (emotion) triggers.
- Avoid drawing conclusions from isolated correlates, and strive for a more encompassing understanding of emotions by considering the broader contextual factors.

3) Species variation: Correlates of emotion can exhibit wide variation between species due to various factors, including their unique evolutionary pressures, ecological contexts, and simply morphological differences. It is crucial to recognise the specific behavioural repertoire of each species and understand how they communicate and express emotions.

- Consider the natural behaviour and social dynamics of each species to ensure accurate interpretation.
- Recognise potential differences also within a species, such as variations between different populations or individuals.

Other methods for analysing facial behaviours

Grimace Scales (GS) have been developed to measure pain based on certain pre-selected facial changes deemed important for pain assessment [121,122]. Originally developed for mice [123], GS have been adapted for various species, including horses [124], sheep [125], rats [126], rabbits [127], and piglets [128]. However, when compared with FACS, GS may be less comprehensive (small number of movements), lack between-species homology (based on appearance changes only, not muscle anatomy), lack reliability between coders (no certification needed), and may not be exclusively coding pain cues (e.g., Andersen's talk reported failure to apply these scales possibly due to confounding factors with fear and stress, **Table S1 in SI**). Geometric Morphometrics (GM) is another analytical method, traditionally used to measure static biological shapes

such as bones, organs, and other structures [129]. Recently it was adapted to investigate facial behaviours, by attributing specific landmarks to animal faces to quantify changes in facial shape [130]. GM reduces potential sources of bias in human-based systems and allows for the consideration of variability between individuals, species, and facial morphology. Due to its early adaptation for measuring facial expressions, further research with this method is however needed.

Advantages and limitations of using AI to automate the detection of animal emotion

1. Advantages:

- **Bypass or reduce biases**: As we detailed in previous sections and **Table S1 in SI**, humans have a range of unconscious, automatic, cognitive processes based on innate mechanisms (e.g., detecting faces and emotions even in objects), as well as a conscious tendency to predict, categorise, and explain visual social information. Whilst scientific training impacts these processes, it does not fully avoid observer biases [131], so AI can help in this.
- **Generate novel insights**: AI systems can identify behaviour cues or features that were not previously considered by human observers, or that the human perception cannot detect reliably. As this is a fairly novel area of research and we still don't fully understand which cues are displayed by each species and in which contexts, AI systems can help identifying these.
- **Allow for multimodality and help explain complexity**: Typically, studies of emotion cues tend to be unimodal (with predominant focus on visual and auditory cues), but emotion is multimodal and there are many streams of data that can be analysed concurrently. This task is nearly impossible to do manually, both in terms of coding, analysis, but also interpretation. AI systems can be crucial in this regard.
- **Reduce time and resource allocation**: AI systems, once fully developed, promise to be faster than the very time-consuming manual coding, and will be more standardised and self-reliable (as machines are always consistent

with themselves) and between studies and research groups. With growing coded data sets (manually and with automated systems), big data analysis in the field of animal emotion will become a possibility.

2. Challenges and current limitations:

Analysing visual cues such as facial and bodily expressions in animals, presents unique challenges and limitations not seen in the human domain:

- **Morphology**: Animals vary in their external morphology much more than humans in relevant categories for visual detection, regarding for example, shapes and colours, even sometimes within the same species.
- **Data collection protocols**: These may immensely vary in different studies regarding the recording equipment used and its angle, environment (e.g., laboratory [92] or in-the-wild setting [93], breed [132], and sex [119]).
- **Data labelling and ground truth**: This is a stage of research where various types of biases can be introduced into the data on which AI models are then trained. In the human domain, self-reporting is often used for establishing ground truth in emotion research. Additionally, employing actors to exhibit emotions is a prevalent method for data collection and labelling in emotion studies. These methods are unfeasible when studying animals, and behaviours in animals are still not well understood, which makes the task of establishing ground truth much more complicated. Some AI projects integrate citizen science in order to cheaply acquire a large amount of data labelling done by the general public, but this is also subject to lack of expertise, training, and may potentially introduce errors. We heard a similar issue in Matsumoto's talk (**Table S1 in SI**), whose data had to be relabelled by experts that were knowledgeable about macaque anatomy (e.g., hidden joints). Furthermore, whilst in some cases, people labelling images are extensively trained and require a certification (e.g., FACS), this is not the case for most data sets (i.e., no quality control is done for the coders' labelling). Finally, most of the automation work with animals, classifies data sets based on a few assumptions: 1) either there

is an emotion displayed or there is not, even when based on more graded scales such as GS for pain assessment (e.g., classifiers discriminate only pain vs. non-pain); 2) there is only one emotion displayed in each context at a certain level, i.e., it excludes more nuanced and naturalistic mixed responses (e.g., pain and fear, as we heard from Anderson's talk - **Table S1 in SI**); 3) there must always be some kind of display indicative of the internal response (i.e., ignores personality and individual variations, or cases in which the absence of changes may be the actual indication of the emotion, such as in the freeze response or typically in prey species that tend to display less overtly negative cues, also featured in Anderson's talk - **Table S1 in SI**); 4) there is no instantaneous variation on the emotion cues or intensity displayed (reported not to be the case in Neethirajan's talk - **Table S1 in SI**).

- **The lack of data sets**: Creating large and diverse databases is crucial for AI development, yet sharing sensitive data on animal behaviour poses several challenges. For instance, videos of animals in cages or following veterinary procedures can be misinterpreted or misused if taken out of context, leading to ethical concerns and potentially inciting backlash against researchers. Additionally, often humans (e.g., companion animal guardians, animal caretakers, researchers, etc.) are present in these recordings and they may perform actions that are not the intended goal of the recording (e.g., private conversations, see [133] for further discussion). Not only issues related to data privacy, but what kind of informed consent owners of the animals gave, which usually does not include AI uses. Finally, many journals make it compulsory or highly advisable to publish the data sets, which may be problematic.
- **Difficulty in capturing the complexities of behaviour**: whilst AI may in many cases help capturing and interpreting complex animal behaviour, such as emotion cues, the opposite may also be true. This may stem both from incomplete data sets (e.g., a single behaviour has many variations but not enough examples) or due to the methods applied. In addition, animals more often than not, present large degrees of movement,

sometimes getting out of view, whilst humans tend to sit or remain still in most situations.

- **Failure to detect subtle or naturalistic behaviour**: In human facial expression research, automated emotion perception systems can easily detect and categorise emotions from stereotypical, exaggerated expressions, but tend to struggle to categorise emotion from subtler and more realistic expressions [134]. Some commercial (e.g., iMotions/Affectiva, [135]) and research [136,137] applications seem to be capturing more subtle facial movements with higher success, but in animal research this has not yet been achieved.

Table S3: A list of human biases (mostly unconscious bias, i.e., automated judgement of a situation based on past experiences, background, knowledge, etc.) that may affect selection, definition, classification, and/or interpretation of animal behaviours, that may also be relevant within the field of automation of animal behaviour research. Adapted from [60,61].

<table>
<tr><th>Type of bias</th><th>Definition</th><th>Examples in animal behaviour research</th><th>Suggestions for bias elimination or minimisation</th></tr>
<tr><td>Attentional blindness</td><td>Perceptual visual narrowing which ignores large changes in the environment or very salient objects due to attentional focus on part of the visual field or a particular event that may be cognitively demanding.</td><td>- Miss changes in body postures because human observers tend to automatically focus on the face.</td><td>- Enhance the observer's ability to notice and record all relevant details, e.g., through observer training and regular calibration sessions, systematic observation protocols, and blinded coding with observers unfamiliar with study aim.</td></tr>
<tr><td>Anthropocentrism</td><td>Human-centric viewpoint, that often leads to the interpretation of animal behaviour primarily in terms of human values and experiences.</td><td>- Attribute (only) “human” emotions to animals (e.g., guilt).
- Fail to acknowledge emotions not part of the human cultural/social experience (e.g., positive anticipation).</td><td rowspan="2">- Employ critical anthropomorphism: use the human emotions to formulate scientific questions and try to understand animal emotions, whilst simultaneously apply a psychobiological approach [120].
- Consider more parsimonious explanations: While a behaviour might indicate social bonding or a learned response to receive rewards, attributing it to complex “human” emotions can lead to misinterpretations of the dog's motivations and cognitive abilities.</td></tr>
<tr><td>Anthropomorphism</td><td>Attributing human characteristics, emotions, and intentions to animals.</td><td>- Interpret a dog's actions (e.g., bringing its owner a toy) as an expression of love in human terms.</td></tr>
</table>

Objectification	Opposite to anthropomorphism, when feelings, intentions or thoughts are denied to exist in other individuals, viewing them as objects.	- When animals are viewed through a Cartesian perspective, with only machine-like responses to a stimulus.<br>- Animals used for food, in which humans first view individuals as “animal category”, but then view the same animal as “food category” so it becomes an object and no ethical considerations are employed.	- Collaborate with experts from various fields such as psychology, neuroscience, and ethology to provide a more rounded understanding of animal behaviour.<br>- Emphasise research that demonstrates the presence of neurological structures and physiological processes in animals that are associated with emotions, cognition, and maybe even consciousness. E.g., brain anatomy, neurochemistry, and neural activity patterns.
Cognitive dissonance	Conciliation of two pieces of contradictory information to reduce psychological discomfort/stress. Contradictions are usually between a person’s beliefs or expectations and reality, which people tend to want to resolve.	- Having different opinions/taking different actions about different individuals from the same species, for example, by making a distinction based on personal feelings (i.e., how much humans care about an owned dog vs a dog in a shelter).<br>- Some species are viewed as objects (e.g., for food, such as farm animals), whilst others are family (e.g., companion animals).	- Develop and promote consistent ethical frameworks that apply to all animals based on their biological characteristics, regardless of the labels attributed by humans based on their beliefs or expectations. For example, ethical frameworks should be based on capacity for suffering and sensorial systems, rather than a person’s belief or expectation about a species utilitarian role.

Confirmation bias	Favour information that confirms one's beliefs or hypotheses [138], which can affect observers and subjects, and works both to confirm one's own beliefs, ideas, and expectations and to reject or disconfirm opposite outcomes.	- E.g., test hypothesis only that will confirm the expected outcome.<br>- E.g., cherry-picking (only cite or mention previous studies that confirm the outcome and ignore the ones that contradict the outcome).	- Blinding everyone involved in the study to its conditions and goals.<br>- Properly training observers.<br>- Testing inter- and intra-observer reliability.<br>- Randomly assigning observers to different groups or treatments.<br>- Use precise and unambiguous operational definitions of behavioural categories.
Observer bias	Having strong preconceptions or a vested interest in the outcome, when the underlying data are ambiguous and/or when the scoring method is subjective [83].	- By holding the belief that chimpanzees have empathy like humans, the researcher might unconsciously interpret various actions (e.g., food sharing) as clear indicators of empathy. This observer bias can lead to an overemphasis on behaviours viewed as empathetic, potentially overlooking other explanations such as reciprocal actions. Consequently, the study might present a skewed understanding of chimpanzees' social dynamics.	

Publication bias	Pressure to publish novel, confirmatory, and statistically significant results, may result in overfit models or p-hacking (when researchers collect or select data or statistical analyses until nonsignificant results become significant) [139,140].	- A significant positive effect of a study is more likely to be published.<br>- Conversely, no significant differences or less impressive outcomes are less likely to be published.<br>- This leads to an over-representation of positive results in the literature.	- Publish negative results or failed methodologies as often as positive results.<br>- Pre-register experimental and statistical protocols.<br>- Avoid p-hacking [141].<br>- Publish preprints.<br>- Ensure (with the future Journal) beforehand that the study will be published independently of the results.<br>- Multi-lab replication efforts, e.g., ManyDogs, ManyPrimates projects [74,75].
Sampling bias	When only some populations/individuals are sampled and these are not representative of the species the claims are being made about. STRANGE framework (adapted from the WEIRD framework for human sampling bias [70]): It stands for: Social background; Trappability and self-selection; Rearing history; Acclimation and habituation; Natural changes in responsiveness; Genetic make-up; and Experience. STRANGE-related biases can	- E.g., test only some dog breeds, overlooking that breed-specific features may influence behaviour. The findings may not accurately apply to other dog breeds with different characteristics. This leads to a sampling bias, limiting the generalisability of the study's conclusions across diverse dog breeds.<br>- E.g., test only captive or only wild individuals of a species.	- Employ the 3D approach: design, declare, discuss, which includes a range of possible solutions and considerations when at each step of a research study (e.g., [72]).

	influence which animals are sampled for testing, the extent to which they participate in experiments and, importantly, the behaviours that they exhibit during trials [71].		
Apophenia	The tendency to perceive meaningful connections between unrelated things. Includes clustering illusion (overestimation of clusters in large samples of random data), illusory correlation (spurious correlations) and pareidolia (perceiving random stimuli as a significant object).	- Clustering illusion: if a behaviour is observed a few times, it may be erroneously considered a pattern;<br>- Illusory correlation: assuming correlated variables as having a causal relationship;<br>- Pareidolia: seeing faces in objects.	- Consider the possibility of these biases when interpreting the results.
Perceptual biases	Natural perceptual processes that usually aid in making sense of the world by filtering information sensed that is not relevant for the task at hand.	- E.g., configural vs. holistic processing of faces – i.e., people do not see facial expressions with its individual AUs, but instead process the whole face make quick judgements on emotions, intentions, communication, etc.	- Acknowledge these biases and acquire training to counteract it. E.g., for facial expressions: FACS training [28,29].<br>- Multidisciplinary teams.<br>- Use detailed and validated observation protocols.

Table S4: A list of biases in AI that may affect selection, labelling, classification and/or interpretation of data sets used for visual classification, detection or recognition of animal behaviour. These biases are not mutually exclusive and may overlap in some situations, but are here defined with the aim of highlighting the issues that can derive from each one. Partially adapted from [61].

**Type of bias**	**Definition**	**Examples in AI applied to animal behaviour**	**Suggestions for bias elimination or minimisation**
Availability bias	Use of the most readily available data (e.g., public databases) or through search engines.	- Use of public databases that can be skewed towards more commonly studied species or particular geographic regions.<br>- Development of AI models based on data sets that are most prominent in search engine results, potentially overlooking less popular but relevant data.	- Evaluate the quality and representativeness of data, regardless of its source.<br>- Assess whether the data adequately covers the relevant diversity of species, behaviours, and environments.<br>- Reporting the sources of data and its limitations.<br>- Continuously review and update AI models as new and more diverse data becomes available, ensuring the models remain accurate and representative over time.
Exclusion bias	When data collected excludes a particular group of individuals.	- Focus on popular breeds of dogs.	- Use stratified sampling to ensure that all relevant subgroups are represented, in proportion to their occurrence in the population.<br>- Analyse the representativeness of the sample and address any limitations in the research findings.<br>- Clearly state if the results are specific to a certain group and avoid overgeneralisation.

Capture bias	Related to the way the images or videos are recorded.	- Exposure, type of camera and lens, framing of the individual as always in the centre, only very good quality of image, low FPS.	- Consider if certain standards for the videos or recording methods may be excluding certain individuals or behaviours.
Contextual bias	Association between a group of individuals and a specific environment or visual background.	- Farming animals (see main text Section 5., subsection "*Are These Biases Also Introduced in the Data Sets from Which AI Learns?"*).<br>- Pain/no-pain studies in which pain individuals are recorded in clinical settings and non-pain at home.	- Ensure that each group of animals is observed in a variety of settings. E.g., observe both pain and non-pain groups in both clinical and home environments.<br>- Replicate studies in different settings to validate findings. If a behaviour is consistently observed across various environments, it is less likely to be a product of contextual bias.<br>- Report the contexts in which observations were made and its potential limitations.
Negative set bias	When the negative/neutral class of images (e.g., non-pain or "neutral"/baseline face) is not representative enough.	- E.g., individuals in a non-pain category, may be expressing many other visual/emotion cues.	- Ensure that a non-pain face is indeed a neutral face, i.e., with absence of visual cues.<br>- Include a diverse class of "non-pain" faces as a baseline.
Automation bias	Tendency to depend excessively on automated systems which can lead to erroneous automated information overriding correct decisions.	- E.g., pain/no-pain classifications with generally low accuracy excessively trusted over lengthy veterinarian assessments.	- Apply trained observers manual checks and quality control of automated classification results.

Pro-innovation bias	The tendency to have an excessive optimism towards an invention or the innovation's usefulness throughout society, while often failing to identify its limitations and weaknesses.	- Excessive optimism about AI capabilities and results, while ignoring its limitations, especially when compared with human behaviour tracking, such as degree of movement, unpredictability of movement, etc.	- Acknowledge limitations of AI and increase data sets with appropriate variability for animal behaviour.
